# Oxidative Stress in the Hypothalamus: the Importance of Calcium Signaling and Mitochondrial ROS in Body Weight Regulation

**DOI:** 10.2174/157015912804143496

**Published:** 2012-12

**Authors:** Erika Gyengesi, George Paxinos, Zane B Andrews

**Affiliations:** 1 Neuroscience Research Australia, Barker Street, Randwick, New South Wales, Australia; 2 The University of New South Wales, Randwick, New South Wales, Australia; 3 Department of Physiology, Faculty of Medicine, Monash University, Melbourne, Victoria, Australia

**Keywords:** Oxidative stress, Reactive oxygen species, NPY, AgRP, POMC, Ghrelin, Leptin, Mitochondrial respiration, Hypothalamic appetite control.

## Abstract

A considerable amount of evidence shows that reactive oxygen species (ROS) in the mammalian brain are directly responsible for cell and tissue function and dysfunction. Excessive reactive oxygen species contribute to various conditions including inflammation, diabetes mellitus, neurodegenerative diseases, tumor formation, and mental disorders such as depression. Increased intracellular calcium levels have toxic roles leading to cell death. However, the exact connection between reactive oxygen production and high calcium stress is not yet fully understood. In this review, we focus on the role of reactive oxygen species and calcium stress in hypothalamic arcuate neurons controlling feeding. We revisit the role of NPY and POMC neurons in the regulation of appetite and energy homeostasis, and consider how ROS and intracellular calcium levels affect these neurons. These novel insights give a new direction to research on hypothalamic mechanisms regulating energy homeostasis and may offer novel treatment strategies for obesity and type-2 diabetes.

## INTRODUCTION

The hypothalamus is a critical region that regulates appetite, body weight and glucose homeostasis. Due to its anatomical localization, it is in a position to directly sense and integrate metabolic information from the periphery and then dictate neural output commands to the corresponding tissues and organs, accordingly. Within the hypothalamus there are several important nuclei regulating appetite and body weight, including the lateral hypothalamic area (LH), the paraventricular (Pa), the dorsomedial (DM), the ventromedial (VMH), and the arcuate hypothalamic nuclei (Arc). Among these areas, the Arc has received most attention and its role in appetite control is fairly well described and defined. In appetite and body weight control, two populations of hypothalamic neurons within in the Arc have mutually antagonistic functions [1]. Activation of neuropeptide-Y (NPY)/agouti-related protein (AgRP) containing neurons results in increased food intake, while the activation of pro-opiomelanocortin (POMC) neurons suppresses appetite [2, 3]. In the Arc, almost all POMC containing neurons also contain cocaine-and-amphetamine responsive transcript (CART), which serves as an anatomical marker and functions as an anorexigenic signal molecule [4, 5]. Within POMC neurons, α-MSH is the critical anorexigenic peptide produced by posttranslational processing of POMC. Both the AgRP/NPY- and POMC/CART - containing neurons contain gamma amino butyric acid (GABA) as their main neurotransmitter [6-9] and project to strongly overlapping areas, including the Pa and the LH and DM [10-12]. POMC neurons contain GABA or glutamate [13, 14], however the precise role of GABA or glutamate transmission from POMC neurons on feeding circuits remains unclear. In their recent and detailed experiments, Dicken *et al*., (2012) elegantly showed that besides the slow and long lasting synaptic actions typical in the case of neuropeptides, POMC neurons are also rapidly regulate both their own activity *via *auto regulation and the activity of downstream neurons [13, 15]. However, recent optogenetic experiments do not support an autoregulatory role of POMC within the Arc [15].

Eukaryotic cells under normal conditions have the ability to regulate the production of reactive oxygen and nitrogen species (ROS/RNS) by controlling the balance between the reduction and oxidation (redox) processes. ROS are chemically reactive molecules and free radicals that contain oxygen, such as peroxides derived from molecular oxygen [16]. At low cellular levels ROS/RNS act as signaling molecules, however at higher concentrations ROS/RNS react with lipids, proteins, DNA/RNA and cause cellular damage to organelles, particularly mitochondria [17]. An imbalance in redox processes causes the build up of ROS/RNS, which eventually contributes to cell damage. Increased levels of ROS/RNS are directly linked to neurodegenerative diseases, such as Alzheimer’s diseases, Parkinson’s diseases, and other diseases like schizophrenia, bipolar disorder, myocardial infarction, and neuropathy in type-2 diabetes mellitus [18-20]. Many antioxidative enzymes and processes in mitochondria and cytoplasm regulate the amount of ROS/RNS produced in cells [21] and act to balance the redox process. Antioxidants remove ROS/RNS by scavenging radicals, decreasing their production, thus preventing oxidative damage. Such scavenger antioxidants are coenzyme Q10, vitamin C and E, and glutathione. Antioxidant enzymes, such as superoxide dismutase (SOD), glutathione peroxidase (GPX) and catalase neutralize ROS including peroxides and superoxide. Some proteins also function as antioxidants by binding ROS/RNS, e.g. acute phase proteins such as albumin, transferrin, haptoglobin and ceruloplasmin [22]. These antioxidant systems protect against the deleterious actions of increased ROS/RNS molecules.

Oxidative stress in neurons increases intracellular Ca^2+^, increases lipid peroxidation, and releases excitatory amino acids from presynaptic buttons (such as glutamate). The role of Ca^2+ ^in cytotoxicity is well known, however the exact mechanisms are still to be clarified [23, 24]. The maintenance of the intracellular Ca^2+^ homeostasis is strongly dependent on precise temporal and spatial control of Ca^2+^-ATPase activity in the plasma membrane, Ca^2+^ uptake into the intracellular stores and the effectiveness of calcium binding proteins (CBPs) in the cytosol [25-27]. These mechanisms are critical, as a shift in the intracellular cation levels, in particular excessive Ca^2+^ accumulation, induces oxidative stress [28].

Moreover, extra and intracellular Ca^2+ ^ion concentrations affect the Ca^2+^-ATPase activity, the tricaboxylic acid cycle and oxidative phosphorylation. When stimulated mitochondria consume more oxygen, which in turn results in more ROS production. Voltage-gated Ca^2+ ^channels are expressed throughout the entire hypothalamus, contributing to the increased cytoplasmic Ca^2+^ levels in sensitive neurons [29]. Alteration of voltage-gated Ca^2+ ^channels by ROS can lead to cell death by apoptosis or excitotoxicity [30]. It is well known that Ca^2+ ^entering the cells leads to elevated oxidative stress in the mitochondria, however the connection has not been thoroughly examined yet.**

This review examines the role of ROS in NPY/AgRP and POMC neurons in hypothalamic processes that regulate energy and glucose metabolism. The accumulated evidence listed below illustrates that ROS play an important physiological role in hypothalamic neurons controlling energy metabolism and glucose homeostasis. In addition, increased intracellular Ca^2+^ level is likely a contributing factor to increase ROS production in these neurons.

## HYPOTHALAMIC ROS REGULATES GLUCOSE AND FATTY ACID SENSING

In recent years, abundant research demonstrates that elevated ROS production causes cellular dysfunction and death, whereas normal ROS levels produced in mitochondria are required as a vital physiological sensor for hypothalamic glucose and fatty acid sensing [31, 32]. The ability of hypothalamic neurons to sense glucose is crucial to maintain body weight and control obesity [33]. Glucose sensing at the cellular level in the Arc is cell-type specific. While POMC neurons utilize glucose as their main fuel, NPY/AgRP neurons are inhibited by high glucose level and may use free fatty acids as their main metabolite [34-36]. Negative energy balance, for example during fasting, is characterized by hypoglycemia and activates NPY/AgRP neuronal firing rate. Interestingly, ROS production during this phase is not significantly increased [34]. On the contrary, POMC neurons are more active in response to feeding or during positive energy balance and use glucose as their main fuel. Under normal chow fed conditions POMC neurons produce significantly more ROS compared to NPY/AgRP neurons [34], implying that metabolic status differentially regulates ROS production in these neurons. Indeed, ROS production needs to be buffered to maintain homeostasis, however the nature of this ROS buffering system in POMC or NPY/AgRP neurons remains unknown. Because these neurons respond differently to positive or negative energy balance, it is likely that NPY/AgRP and POMC neurons possess either 1) unique molecular machinery and/or 2) unique upstream sensing mechanisms that activate cellular ROS buffering systems.

The cellular pathways through which Ca^2+^ increases ROS production during glycolysis has been examined by Hernandez-Fonseca *et al*., (2008) in hippocampal primary neuronal cell cultures. Their results confirmed that hypoglycemia associates with increased ROS production and excitotoxicity. Further, Ca^2+ ^influx through *N*-Methyl-D-aspartate (NMDA) receptors, which is one of the ionotropic glutamate receptors, plays a crucial role in ROS production during moderate energy depletion [37]. NPY/AgRP neurons are located close to the median eminence and capable of sensing nutrient and hormone concentrations in the blood. Kuo *et al*., (2011) recently examined how redox signaling *via *ROS contributes to nutrient sensing and appetite regulation [38]. In the same study, they investigated the effects of phenylpropanolamine (PPA), an appetite-suppressing sympathomimetic agent, on both NPY and POMC neurons. PPA induces initial weight loss during a 4-day treatment period in rats. This anorexigenic effect is mediated by catecholamine release that has an inhibitory effect on hypothalamic NPY neurons [39]. In the same study, they found that the PPA not only suppresses appetite but also increases endogenous antioxidants, such as SOD and glutathione S-transferase [38] and increases POMC expression. At the same time, PPA reduces NPY expression, which collectively results in decreased food intake and weight loss. Their findings confirmed that ROS plays a role in appetite control by anticipating in both lipid and glucose sensing [40, 41].

Protein kinase C (PKC) is a common intracellular signaling protein that is particularly sensitive to redox state changes. PKC- λ is a Ca^2+^ independent, but triacylglycerol dependent, atypical PKC isoform. Kuo *et al*., (2011) showed that PKC- λ mediated the appetite-suppressing effect of PPA by increasing oxidative stress. The increased ROS production stimulated POMC activity and inhibited NPY activity [42]. These results also indicate that ROS-induced signaling mechanisms are more sensitive in POMC neurons.

Benani *et al.,* (2007) showed that increased levels of plasma triglyceride, which is an endocrine marker of positive energy balance, resulted in local and rapid production of ROS in the brain. In parallel, increased mitochondrial O_2_ consumption also triggered ROS production. The authors found that the elevated hypothalamic ROS modified the redox state of oxidized and reduced glutathione but failed to cause oxidative stress measured by dichlorofluorescein fluorescence *ex vivo*. These authors concluded that acute hypertriglyceridemia triggered hypothalamic ROS production due to the increased mitochondrial activity [32].

These findings support the notion that ROS levels influence NPY/AgRP and POMC neuronal function. Redox signals and ROS respond to metabolic signals and influence the ability of NPY/AgRP or POMC neurons to sense fuel availability, regulate food intake and body weight.

## MITOCHONDRIAL RESPIRATION AND OXIDATIVE STRESS IN THE HYPOTHALAMUS

Mitochondrial dysfunctional causes oxidative stress, as mitochondria generate, and are targeted by, ROS. During normal cellular respiration, O_2_ is reduced to H_2_O in mitochondria by oxidative phosphorylation, which produces energy in the form of ATP for cell function. During these reactions, electrons travel through the mitochondrial electron transfer chain (ETC) that includes complex I (NADH uniquinone oxireductase), complex II (succinate ubiquinone oxireductase), complex III (ubuquinone-cytochrome c oxidase), complex IV (cytochrome c oxidase), complex V (ATP synthase), as well as ubiquinone and cytochrome c. The complexes I to IV work to establish a H^+^ gradient across the inner membrane of the mitochondria that creates the electrochemical energy gradient to drive complex V to synthetize ATP. The reduction of O_2_ to H_2_O produces several ROS molecules, including H_2_O_2_, O_2_^-^ and OH●. Complex I and III produce ROS in the highest capacity [43, 44]. Besides the ETC, the NADPH oxidase and ATPase are also an alternative source of O_2_^- ^in the central nervous system.

The role of complex I in both anorexia nervosa and hypothalamic degeneration was further examined by Lindfors *et al*., (2011) taking advantage of the *anx/anx* mouse model. They provided evidence for the mitochondrial dysfunction in the hypothalamus by measuring mitochondrial respiration and detected significant reduction in complex I-driven respiration, while complexes II-V remained intact [45]. The authors hypothesized that the symptoms of anorexia could be related to defective mitochondrial oxidative phosphorylation at complex I [46]. Complex I deficiency arises from mutations in nuclear DNA-encoded subunits and complex I is a major contributor to ROS production. Therefore, defective complex 1 activity in *anx/anx* mice presumably leads to increased hypothalamic ROS production, which may have a direct effect on hypothalamic circuits regulating satiety, resulting in a chronic state of anorexia. This hypothesis, however, remains to be tested.

Only recently, studies have begun to explore the role of ROS specifically in the NPY/AgRP and POMC/CART neurons. Diano *et al.,* (2011) revealed that a decrease in ROS production reduces POMC cell activation and increases NPY/AgRP cell activation, which in turn promotes feeding. These results highlight that sufficient ROS production is required to maintain POMC neuronal function and to reduce feeding, which is consistent with studies by Kuo *et al*., (2012) presented above. This study also showed that ROS scavenger treatment increased c-fos expression in NPY/AgRP neurons and an elevated food intake in normal mice. ROS scavenger treatment also lowered c-fos expression and initiated a significantly higher peroxisome number formation in POMC neurons of diet-induced obese mice, indicating that ROS activates POMC neurons [47]. High fat diet caused peroxisome proliferation and decreased ROS levels in POMC neurons and promoted food intake. On the contrary, suppressed peroxisome proliferation increased ROS levels in POMC neurons. Direct application of ROS into the central nervous system increased POMC neuronal activity and decreased food intake in diet-induced obese mice. These findings suggest that ROS plays an important regulatory role in POMC neuronal function to maintain energy homeostasis. Positive energy balance, which is characterized by hyperglycemia and glycolytic metabolism, activates POMC neurons and generates significant ROS that acts as an endogenous intracellular feedback signal in POMC neurons to control activity. Consistent with this idea, hyperglycemia induces mitochondrial ROS production [48]. In negative energy balance, NPY/AgRP neurons respond to the low levels of glucose and increase neuronal firing rate [49] and potentially use fatty acids as fuel to sustain this firing. Increased neuronal activity is followed by Ca^2+^ influx, which elevates ROS production in neurons [37]. However, NPY/AgRP neurons do not produce and release considerable amount of ROS. These results indicate that NPY/AgRP neurons have unique cellular properties that rapidly and efficiently buffer ROS production or a mechanism that restricts ROS production.

Ghrelin and leptin are two major hormonal regulators of appetite and energy homeostasis. *Ghrelin* is a gut-derived hormone that increases NPY/AgRP activity in the Arc and increases food intake [34]. Ghrelin activates AMP-activated protein kinase (AMPK) in NPY neurons, which inhibits acetyl choline-A carboxylase and fatty acid synthase, and importantly increases mitochondrial respiration and ROS production [50, 51]. Ghrelin increased hypothalamic mitochondrial respiration *via *uncoupling proteins (UCP2), which in turn reduces ROS production and scavenges free radicals, as ghrelin elevated synaptosomal ROS production UCP2 knockout mice, but not in their wild-type counterparts. In situ ROS levels detected by dihydroethidium bromide showed that UCP2 buffers ghrelin-induced ROS production in identified NPY neurons, whereas ghrelin did not affect POMC ROS production [34]. The ROS buffering properties of UCP2 allows ghrelin to sustain NPY/AgRP neuronal activity under conditions of negative energy balance. Interestingly, baseline ROS levels in POMC neurons are higher than NPY/AgRP neurons, suggesting POMC neurons may be more vulnerable to free radical-induced degeneration. Uncoupling proteins have the capacity to reduce ATP and ROS production by promoting fatty acid oxidation and increase mitochondrial biogenesis, hence they also play a role in the rate of aging and life span [52, 53].

The adipose tissue derived hormone *leptin* plays a central role in the regulation of energy homeostasis. Leptin acts *via *the long form of the leptin receptor (LepRb) that is expressed on both NPY/AgRP and POMC/CART neurons in the hypothalamus, mostly in the ventral premamillary (PMV), the DM and Arc nucleus and modulates the activity of both NPY/AgRP and POMC neurons [54]. Whether or not leptin influences ROS production to maintain POMC neuronal function remains to be determined. It is known that leptin depolarizes POMC neurons, however the exact description of the ion channels and currents involved remains to be determined. Using whole cell recording in POMC-EGFP transgenic mice, Qiu *et al*., (2010) found that transient receptor potential (TRCP) channel blockers inhibited the leptin-induced depolarization in POMC neurons, while lanthanum (La^3+^) and intracellular Ca^2+^ potentiated the effect of leptin [55]. Under physiological conditions, extracellular Na^+ ^activates a nonspecific cation current and increases intracellular Ca^2+ ^levels [56].

## ROLE OF CALCIUM/CALCIUM BINDING PROTEINS IN THE HYPOTHALAMUS

The maintenance of the intracellular Ca^2+^ homeostasis depends on the precise temporal and spatial control of Ca^2+^-ATPases activity in the plasma membrane, Ca^2+^ uptake into the intracellular stores, influx through voltage-operated and receptor-operated channels, release from intracellular compartments and the effectiveness of Ca^2+ ^buffering *via *CBPs in the cytosol and the plasma membrane [25-27, 57, 58]. Excessive intracellular Ca^2+^ accumulation causes mitochondrial ROS production and significantly elevates oxidative stress within cells [28]. Neuronal Ca^2+^ is stored either in the endoplasmatic reticulum (ER) or in mitochondria and both organelles contain very high Ca^2+^ concentrations. Ca^2+ ^entry into the mitochondria matrix depolarizes the mitochondrial membrane potential and reduces ATP synthesis [59]. Indeed, excessive Ca^2+^release from these storage organelles indicate cellular damage that eventually leads to cell death [60]. There is also evidence that ROS produced by the mitochondria are directly involved in cell death [61].

Ca^2+^ is a key signaling molecule involved in regulating food intake, body weight and glucose homeostasis. Recently neuronal over-expression of the Ca^2+^/calmodulin dependent protein kinase (CaMKK) was shown to protect mice from high fat diet-induced weight gain, insulin resistance and glucose homeostasis. CaMKK is activated by the change of the intracellular Ca^2+ ^[62-64] and acts as an upstream activator of AMPK, independent of AMP levels. In hypothalamic neurons, CaMKK regulates orexigenic NPY production [65, 66]. Ghrelin activates the ghrelin receptor (GHSR), a seven transmembrane G-coupled protein linked to Gq and activation of these receptors on Arc NPY neurons increases intracellular Ca^2+^ levels and activates CamKK [67]. Activated CaMKK stimulates AMPK pathways, that results in increased ATP generation and fatty acid oxidation, which leads directly to increased ROS production in the cells [68, 69].

Ca^2+ ^plays an important, albeit underappreciated, role in appetite regulation and energy homeostasis through central and peripheral effects. Further study is required to elucidate the role of Ca^2+ ^in defined neuronal populations important for appetite regulation such as NPY/AgRP and POMC. Indeed, CBPs are important regulators of intracellular Ca^2+^ concentrations and hence it is crucial to investigate their role in regulating Ca^2+^ levels in certain neuron populations.

## CALCIUM BINDING PROTEINS IN THE HYPO-THALAMUS

The intracellular Ca^2+^ concentration is approximately 20,000-fold lower than extracellular Ca^2+^, indicating the precise cellular control of cytoplasmic Ca^2+^ levels [70]. Indeed, the resting intracellular cytosolic Ca^2+ ^concentration is around 100nM, which can increase up to 1-2 μM in response to various stimuli in the Arc nucleus [71, 72]. This clearly necessitates efficient and rapid intracellular Ca^2+^ buffering and CBPs represent one such buffering system. CBPs sense and bind to Ca^2+^ ions to maintain intracellular Ca^2+^ homeostasis. Although present in various hypothalamic nuclei, their functional role has not been clarified. Here we describe the hypothalamic expression of CBPs and discuss the functional implication of CBPs on Ca^2+^-induced ROS production and energy metabolism.

Parvalbumin (Pv) is expressed in the ventrolateral tuberal hypothalamus (PV1 nucleus) in rats and mice [73, 74]. However, little is known about the function of these neurons. Interestingly, unlike most Pv containing neurons in the brain that express GABA, these neurons contain the excitatory neurotransmitter glutamate [74]. Glutamate increases ROS production with or without extracellular Ca^2+^ [75]. The activation of ionotropic glutamate receptors leads to Ca^2+^ influx into the cell from the extracellular space, resulting in increased Ca^2+^ level [76]. In addition, activation of the metabotropic glutamate receptors also leads to Ca^2+^ release from intracellular stores [77]. These studies highlight that cells with glutamate neurotransmission require efficient Ca^2+^ buffering properties, such as CBPs. Besides the PV1 nucleus in the hypothalamus, the Arc expresses Pv containing neurons, which show a 10-fold increase in response to estrogen in female mice [78].

Secretagogin (Scgn) is a recently described CBP cloned from pancreatic (-cells and endocrine cells of the gastrointestinal tract [79]. Scgn is a member of the EF-hand family of CBPs containing 3D –motif to bind Ca^2+^ in the cytosol. It is widely expressed in the rodent, primate and human brain, serving as a useful marker for areas in the telencephalon, such as the olfactory bulb, the hippocampus, amygdaloid complex, the basal forebrain and the hypothalamus [80-82]. Within the hypothalamus, Scgn-containing neurons are located in the Arc, the periventricular nucleus, the Pa, and the DMH. In the Arc, we found that approximately 50% of the NPY-AgRP neurons contain Scgn (Gyengesi, unpublished). We also found that Scgn is not co-expressed with POMC in the hypothalamus. These findings indicate that Scgn expression might play an important role in appetite by buffering intracellular Ca^2+^ in NPY, but not POMC neurons, during negative energy balance. Indeed, ghrelin levels increase during negative energy balance and stimulate feeding behavior by increasing intracellular cytosolic Ca^2+ ^concentration, CaMKK and AMPK in NPY but not POMC neurons [6, 51, 83]. Thus, there is a direct requirement to buffer Ca^2+^ in NPY but not POMC neurons to maintain cellular firing during negative energy balance. Moreover, NPY/AgRP neurons generate less ROS production compared to POMC neurons [34]. Because buffering intracellular Ca^2+^ controls ROS production, the lack of Scgn and other CBPs in POMC neurons might contribute to elevated ROS compared to NPY/AgRP.

Calretinin-positive perikarya and neuropil were noticed in the ventral and dorsal portions of the suprachiasmiatic nucleus (SCh), lacking immunoreactivity in the central core of the SCh of the common marmoset [84]. Calretinin immunoreactivity was low in the neonatal SCh and increased with development in the ventrolateral SCh [85].

While it is one of the most commonly distributed CBP in the brain, little is known about the expression pattern and functional role of calbindin D28k (Cb) in the hypothalamic feeding circuit in mice. Based on the Chemoarchitectonic Atlas of the Mouse Brain by Watson and Paxinos, Cb is expressed in the cell bodies of the Pa, DMH, Arc, periventricular and VMH, together with strong neuropil [86].

## SIGNALING MOLECULES AND PATHWAYS IN CONNECTION WITH CA^2+^ AND ROS

AMPK is a serine/threonine protein kinase that senses nutrients and hormones in the hypothalamus [87]. It plays a key role in general growth and metabolism of the cells. Deleting the catalytic subunit (ampkα2) from POMC neurons causes obesity and impaired glucose sensing [88, 89]. CaMKK-β is an upstream regulator of AMPK, which is also involved in the regulation of energy homeostasis [65]. CaMKK-β activates AMPK depending on intracellular Ca^2+^ levels and initiates energy producing mechanisms to restore ATP levels. To further examine the role of CaMKK in the hypothalamic control of appetite, Anderson *et al*., generated neuron specific CaMKK deletions [65]. Deletion of CaMKK-β in NPY/AgRP neurons reduced food intake and body weight, while deleting CaMKK-β in POMC neurons did not affect energy homeostasis or glucose metabolism [65, 89, 90]. Racioppi *et al*., (2012) recently described that genetic ablation of CaMKK2 protected mice from diet-induced obesity, insulin resistance and glucose intolerance. CaMKK2 prevented high fat diet-induced glucose intolerance by attenuating the inflammatory response in adipose [65, 91]. CaMKK2 is responsible for regulating the amplitude of the macrophage inflammatory response, which is induced by excess nutrient or pathogens [91].

A recent study described a presynaptic mechanism involving Ca^2+^ release that maintains NPY/AgRP neuronal firing [49]. Yang *et al.,* (2011) showed that food deprivation increases the number of excitatory inputs on NPY/AgRP neurons that resulted in increased NPY/AgRP activity, which in turn stimulated feeding behavior. Ghrelin increased a presynaptic signaling pathway that induced direct Ca^2+^ release from internal stores *via *the ryanodine receptor. This activated presynaptic AMPK in nerve terminals synapsing onto NPY/AgRP neurons and maintained elevated NPY/AgRP action [49]. Kohno *et al.,* (2011) showed that NPY/AgRP neurons but not POMC respond to the AMPK activator, 5-amino-1-β-D-ribofuranosyl-imidazole-4-carboxamide (AICAR), and increase intracellular Ca^2+^ levels. This study also showed that intracerebroventricular administration of AICAR increased food intake *via *AMPK activation of NPY neurons through Ca^2+^ influx, causing NPY-dependent food intake [72].

Taken together with the fact the POMC neurons do not contain any of the above mentioned, classical CBPs, such as Pv, Cb, Cr or Scgn, it is not surprising that the lack of a CaMKK-β, had little or no effect on POMC glucose or energy homeostasis. Li *et al*., (2010) showed that Ca^2+^ activates AMPK and oxygen free radicals also increase glucose uptake *via *glucose transporter GLUT4 in neurons [92], consistent with the idea that Ca^2+^ and ROS promote neuronal metabolism during negative energy balance when NPY/AgRP neurons are active and POMC neurons are silent. A recent study has also described an additional role of AMPK in mitophagy and mitochondrial biogenesis. Mihaylova *et al.,* (2011) showed that activation of AMPK leads to the degradation of defective mitochondria and at the same time stimulates mitochondrial biogenesis [93]. Thus removing faulty mitochondria and generating new healthy mitochondria will to help combat cell stress by increasing energy production and decreasing mitochondrial oxidative stress. Nishihara *et al.,* (2012) showed that, at least in the rostral ventrolateral medulla of the brainstem, ROS enhance glutamatergic excitatory input [94]. Two new studies showed that excitatory synaptic input in the Arc control NPY/AgRP containing neurons but not POMC [49, 95]. Putting these two findings together, we hypothesize that ROS may indirectly control feeding behavior, *via *increased glutamatergic input on NPY/AgRP neurons. Interestingly, deletion of NMDA receptors, a key route for Ca^2+^ to entry the cells, on NPY/AgRP neurons did not show any response to fasting. This finding suggests that Ca^2+ ^signaling through NMDARs plays an important role to control energy homeostasis.

## PHARMACOLOGICAL ASPECTS OF REDOX REGULATION IN THE FEEDING CIRCUIT OF THE BRAIN

The role of activated ionotropic glutamate receptors in Ca^2+^-induced oxidative stress in neurons was suggested almost 20 years ago, however the exact details of the mechanisms are still not completely understood. Increasing number of articles has been recently published showing that ionotropic glutamate receptors, such as the NMDA receptors, play a significant role in controlling the energy homeostasis and the maintenance of the cytosolic Ca^2+^ levels by forming a major Ca^2+ ^entry system of the plasma membrane [29, 30]. NMDA receptors are responsible for cellular redox states and modified by either reduction, causing receptor potentiation, or oxidation, causing inhibition of receptor function [96]. Ligand binding initiates receptor opening by inducing conformational changes at the extracellular ligand-binding domain. The only redox modulation site on the NMDA receptors is a disulfide bond at the GluN1 subunit. Elimination of this bond by dithiothreitol potentiates the macroscopic current amplitude, increases the frequency and the duration of the channel opening [97-99]. Khan *et al.,* (2004) showed that NMDA receptor activation in the lateral hypothalamus by acute microinjection of l–glutamate or other agonists plays a role in feeding [100]. Molecular and pharmacological inhibition of NMDA receptors in the hypothalamus is mediated by suppressed AMPK activity and lowers glucose production and increases feeding [101]. Several studies showed that AMPK in the hypothalamus regulates energy metabolism by integrating inputs from hormones, peptides, neurotransmitters, and nutrients [102-104]. It is highly likely that glutamate-induced neuronal excitation *via *NMDA receptors mediates the production of ROS. This event is accompanied by Ca^2+ ^influx from the extracellular space, while Ca^2+^ is also released from the mitochondria. Due to an increase in the intracellular Ca^2+ ^levels, cellular swelling occurs and cellular organelles take up Ca^2+ ^to prevent further cell damage and death. However, the initial increase of ROS serves as a signal to prevent cell damage.

## SUMMARY

Appetite is a complex behavior that involves homeostatic mechanisms driven by hypothalamic pathways. This review highlights that ROS levels, controlled by various ionic and fuel signals, play an important role in the hypothalamus. In both NPY and POMC neurons, ROS serve as useful markers in fuel sensing and utilization, but also byproducts of faulty mitochondrial respiration at high levels. Intracellular ROS levels directly control cellular activity of NPY/AgRP and POMC neuronal population and regulate food intake and body weight. At high levels that saturate buffering mechanisms, ROS may initiate cellular degeneration in either NPY/AgRP or POMC neurons and impair energy homeostasis. CBPs play a crucial role in Ca^2+^ buffering and preventing excessing ROS production. Although the exact function and expression of CBPs in hypothalamic neurons is unknown, we hypothesize that CBPs buffer Ca^2+^ and ROS in NPY/AgRP to maintain prolonged firing during negative energy balance.

## Figures and Tables

**Fig. (1):**
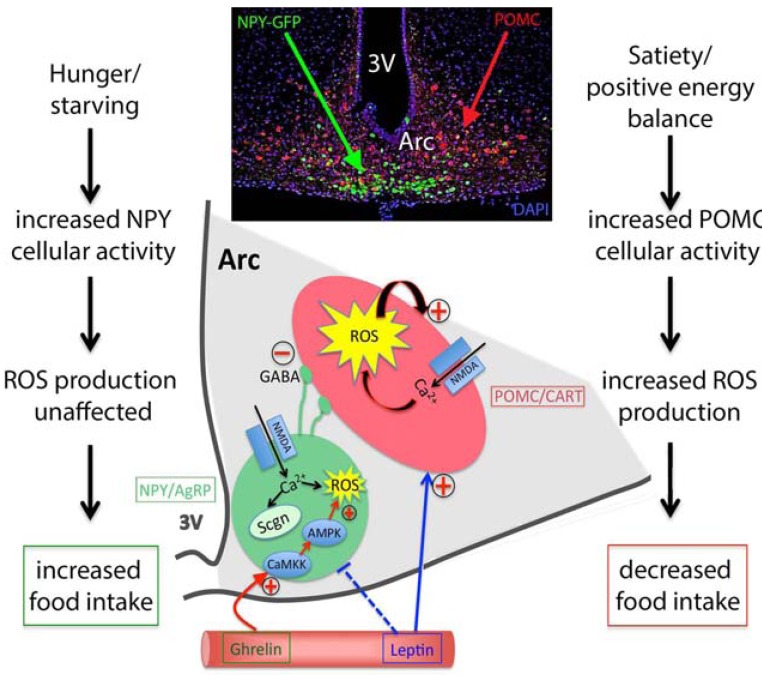
NPY/AgRP and POMC/CART neurons are in close proximity as shown on the inset of Fig. (1)., however they produce opposing actions on food intake and body weight balance. Both ROS and CBPs may play important roles to mediate the differing actions on food intake and body weight balance. In satiety or during positive energy balance, POMC neurons are more active, using mostly glucose as their primary fuel. The increased cellular activity results in increased ROS production and this elevated ROS production also has an autoregulatory effect on POMC neurons, further increasing their activity. These cellular events act to suppress feeding behavior and decreasing further food intake. Under conditions of negative energy balance, such as fasting or calorie restrictions, NPY neurons become more active, using free fatty acids as their main fuel that results in feeding behavior. Secretagogin in NPY neurons potentially buffers excessive intracellular calcium and prevents ROS build up. Hormones in the blood stream, such as leptin and ghrelin, have the ability to increase the activity of POMC and NPY neurons, respectively. Once activated, NPY neurons send GABAergic, inhibitory synapsis directly to POMC neurons, to further strengthen the cellular pathways of food intake.

## ABBREVIATIONS

AgRP: = agouti-related peptide
AICAR: = 5-amino-1-β-D-ribofuranosyl-imidazole-4-carboxamide
AMPK: = 5' AMP-activated protein kinase
Arc: = arcuate hypothalamic nucleus
CaMKK: = calmodulin-dependent protein kinase kinase
CART: = cocaine and amphetamine related transcript
Cb: = calbindin
CBP: = calcium binding protein
Cr: = calretinin
DM: = dorsomedial hypothalamic nucleus
ETC: = electron transfer chain
GPX: = glutathione peroxidase
LepRb: = leptin receptor
LH: = lateral hypothalamic area
NMDA: = *N*-Methyl-D-aspartate
NPY: = neuropeptide-Y
Pa: = paraventricular hypothalamic nucleus
PKC: = protein kinase C
PMV: = premammillary nucleus
POMC: = pro-opiomelanocortin
PPA: = phenylpropanolamine
Pv: = parvalbumin
RNS: = reactive nitrogen species
ROS: = reactive oxygen species
Scgn: = secretagogin
SCh: = suprachiasmatic nucleus
SOD: = superoxide dismutase
UCP2: = uncoupling protein 2
VMH: = ventromedial hypothalamic nucleus
